# Hebrew Digits in Noise (DIN) Test in Cochlear Implant Users and Normal Hearing Listeners

**DOI:** 10.3390/audiolres14030038

**Published:** 2024-05-20

**Authors:** Riki Taitelbaum-Swead, Leah Fostick

**Affiliations:** 1 Department of Communication Disorders, Speech Perception and Listening Effort Lab in the Name of Prof. Mordechai Himelfarb, Ariel University, Ariel 4070000, Israel; 2 Meuhedet Health Services, Tel Aviv 6203854, Israel; 3 Department of Communication Disorders, Auditory Perception Lab in the Name of Laurent Levy, Ariel University, Ariel 4070000, Israel; leah.fostick@ariel.ac.il

**Keywords:** digits in noise (DIN), speech perception, cochlear implants

## Abstract

This study aimed to compare the Hebrew version of the digits-in-noise (DIN) thresholds among cochlear implant (CI) users and their normal-hearing (NH) counterparts, explore the influence of age on these thresholds, examine the effects of early auditory exposure versus its absence on DIN threshold, and assess the correlation between DIN thresholds and other speech perception tests. A total of 13 children with CI (aged 5.5–11 years), 15 pre-lingual CI users (aged 14–30 years), and 15 post-lingual CI users (aged 22–77 years), and their age-matched NH controls (n = 45) participated in the study. Speech perception tasks, including the DIN test, one-syllable word test, and sentence identification tasks in various auditory conditions, served as the main outcome measures. The results indicated that CI users exhibited higher speech reception thresholds in noise across all age groups compared to NH peers, with no significant difference between pre-lingual and post-lingual CI users. Significant differences were also observed in monosyllabic word and sentence accuracy in both quiet and noise conditions between CI and NH groups. Furthermore, correlations were observed between the DIN and other speech perception tests. The study concludes that CI users require a notably higher signal-to-noise ratio to discern digits in noise, underscoring the DIN test’s utility in assessing speech recognition capabilities in CI users while emphasizing the need for a comprehensive test battery to fully gauge their speech perception abilities.

## 1. Introduction

Over the last four decades, cochlear implantation has become the preferred rehabilitation option for children and adults with severe-to-profound hearing loss [1]. Currently, approximately 1,000,000 cochlear implant (CIs) users are registered worldwide [2], and this number is anticipated to increase substantially in the coming years. The success of this procedure due to improvements in the technology of implant devices has led to the broadening of candidacy criteria, including a decrease in the age of implantation in young infants, most of them with bilateral CIs [3,4], and an increase in the age of older adults who receive CI [5]. This also led to the implantation of CIs in candidates with better hearing thresholds and speech perception abilities.

Speech perception in degraded listening situations, such as noisy environments, remains a significant challenge for CI users [6,7,8,9]. CI recipients experience difficulties in noisy environments due to the degraded signal transmitted by the CI device (poor “bottom-up” processing). Therefore, there is a substantial need to evaluate their speech perception abilities, particularly in noisy conditions. However, due to the degraded bottom-up information in these situations, CI users must rely on top-down information, such as semantic and syntactic knowledge. In turn, this adds cognitive function as a confounding factor that can influence the results [10]. The need for linguistic knowledge can also limit the range of participants performing speech-perception-in-noise tests, such as children and nonnative speakers. Accordingly, there is a need for a short and efficient test that will evaluate speech-perception-in-noise while matching the CI users’ age and auditory abilities.

The digit in noise (DIN) test emulates everyday conditions by using speech stimuli embedded in noise but with minimal linguistic knowledge. This test was originally developed as a screening tool for hearing loss; however, it has recently become an acceptable measure of speech perception abilities in adverse listening conditions. The test presents a series of three digits (typically constructed from digits 0 to 9) in the presence of speech-shaped noise constructed from the digits’ spectrum. The listener is then asked to repeat the digits in the correct order. The intensity level of the digits varies amidst the noise to assess an individual’s ability to understand speech in increasingly difficult listening conditions. Since the test stimuli are digits, it is appropriate for young children or others with limited language skills as well as for adults who possess advanced language proficiency [11]. The other advantages of this short and efficient test include the ability to change the signal-to-noise ratio adaptively and its applicability in various auditory abilities.

The results of the DIN test have been examined in various languages and populations of CI users and were shown to correlate with the outcomes of other speech perceptions in noise tests [12,13,14]. However, it is unclear whether the DIN test, primarily designed as a screening tool, can be sensitive enough to distinguish between different age groups and detect the onset of deafness, making it suitable for inclusion in the speech perception battery for follow-up in CI centers. Thus, the present study aimed (1) to compare the DIN thresholds between CI users and normal-hearing (NH) peers, (2) to test the effect of age on DIN thresholds, (3) to investigate the impact of early auditory exposure (post-lingual CI users) versus its absence (pre-lingual CI users) on the DIN threshold, and (4) to explore the association between DIN thresholds and other open-set speech perception tests. Due to group differences in age and, consequently, linguistic and auditory abilities, speech perception was assessed in children using monosyllabic words and sentences without background noise, and in adults using sentences with background noise.

## 2. Materials and Methods

### 2.1. Participants

A total of 88 children and adults participated in the study: 13 prelingual CI children and 15 NH peers aged 5.5–11 years, 15 pre-lingual CI users (aged 14–30 years), and 15 post-lingual CI users (aged 22–77 years), and their age-matched NH controls (n = 30). All 43 CI users were recruited via associations for hearing-impaired individuals, whereas the NH controls were recruited via social media advertisements. The inclusion criteria for all participants were as follows: (a) native Hebrew speaker, (b) no reported cognitive or attention deficit disorders, and (c) at least 12 years of education for all adult participants and the children’s parents. These inclusion criteria were assessed using self-report questionnaires completed by adult participants or the children’s parents. The study was approved by the institutional ethics committee and all adult participants and the children’s parents provided informed consent before the experiment commenced. Participants received an equivalent of 25 USD as compensation for the time spent participating in the study.

#### 2.1.1. CI Groups

Three groups of CI users participated in the present study. One group was of children with CI and included 13 CI users aged 5.5–11 years (mean, 7.6 years). All the children had bilateral severe to profound hearing loss before 2.5 years of age, and their age at implantation (first or only CI) ranged between 0.75 and 6.5 years (mean, 2.4 years). Eight children used bilateral CIs, and five used bimodal amplification (CI+ hearing aid [HA]); eight used cochlear devices, and five used advanced bionic devices. A second group included 15 pre-lingual CI users aged 14–30 (mean, 22.6 years). These pre-lingual young adults had bilateral severe to profound hearing loss before 1.5 years of age, and their age at implantation (first or only CI) ranged between 1 and 8 years (mean, 2.95 years). Thirteen young adults used bilateral CIs, one used bimodal amplification (CI+HA), and one used only one CI; 14 used a cochlear device, and one used an advanced bionics device. The third group included 15 post-lingual CI users aged 22–77 years (mean, 51 years), and their age at implantation (first or only CI) ranged between 17 and 73 years (mean, 46 years). All the post-lingual CI users had bilateral severe to profound deafness after eight years of age. Three adults used bilateral CIs, ten used bimodal amplification (CI+HA), and two used only one CI; six participants used the cochlear device, four used the advanced bionics device, and five used the Med-El device. The individual demographic information and background data for each CI user are presented in Appendix A.

#### 2.1.2. NH Groups

The NH participants were matched to the participants in the CI groups in terms of age (within 6 months), sex, and level of education. The additional inclusion criterion for the NH group was a mean pure-tone average (PTA; 0.5, 1, 2, 4, and 8 kHz) of less than 20 dB HL in both ears for participants under 60 years of age and less than 30 dB HL for participants aged 60 years or older, as verified by an audiology student.

### 2.2. Speech Perception Measures

Speech perception was measured using the Hebrew DIN test, monosyllables (Hebrew Arthur Boothroyd [HAB] test), and sentences (the HeBio test for adults and sentences for children).

#### 2.2.1. Hebrew Digits in Noise (DIN) Test

This test was originally developed in the Netherlands by Smits et al. (2004) [11]. It included 24 broadband homogeneous digit triplets which were used to test digit recognition. In the Hebrew version of the DIN test, the digits are pronounced by a female speaker and presented in long-term average speech spectrum (LTASS) masking noises [11]. The development and adaptation of the Hebrew version of the DIN test were similar to that of the Dutch version and followed its stages [15]. In the adaptive procedure, the overall intensity level was kept constant at 65 dBA and the first digit triplet was presented at 0 dB signal-to-noise ratio (SNR). The intensity of the triple digits varied adaptively according to the correctness of the response, following a standard one-up, one-down procedure with a step size of 2 dB. The outcome SRTn (dB) was the average presentation SNR of the final ten reversals. All three digits had to be pressed correctly for the triplet to qualify as correct (four triplets for training at a fixed SNR of 0 dB and 20 for testing).

#### 2.2.2. HAB Words

The Hebrew version of the Arthur Boothroyd Words Test (HAB) [16,17] is composed of 15 lists of ten meaningful, one-syllable consonant-vowel-consonant phonetically balanced Hebrew words (i.e., in each list, every consonant appears once and every vowel appears twice). For each participant, two lists of ten words were presented. The participants were instructed to repeat each word they heard and to guess if they were unsure. The results were manually recorded using the experimenter and expressed as a percentage of correct words.

#### 2.2.3. HeBio Sentence in Quiet

This test included 33 sentence lists. Each list included 20 sentences, with five sentences from each of the four speakers (two men and two women). Sentences were composed without restrictions on complexity, vocabulary, or phonemic content. They did not include names of people, places, or objects. The mean sentence length was 6.12 with a standard deviation of 1.70. The sentences were aimed at reflecting examples of the daily discourse of adults and were meaningful, yet not predictable. They were scored according to the number of words that were repeated correctly out of the total number of words in the sentence. Errors related to tense, person (number and sex), stem, or verb pattern were counted as incorrect, but reporting the correct words in a different order than in the original sentence (e.g., reporting ‘the dog I bought’ instead of ‘I bought the dog’) did not affect the scoring. The intelligibility score for each sentence was expressed as a percentage of correct words.

#### 2.2.4. Adaptive HeBio in Noise

In the adaptive paradigm, the intensity of the speech-shaped noise (SSN) varied adaptively using a one-down-one-up procedure to target the SNR at which 50% of the speech was identified correctly, that is, the SRTn. The initial SNR was +10 dB, with a step size of 5 dB. When participants did not repeat a sentence correctly (according to the criterion), the noise decreased or increased by 2 dB steps. The intensity steps were maintained at 2 dB until the end of the list. The estimated SRTn thresholds for each list were calculated according to the mean SNR of the last ten sentences in each list. The intensity of the speech was kept constant at 65 dB SPL, which was chosen so that the utterances were clearly audible for all participants. The SSN was a steady-state narrow-band noise that matched the existing speech signals and was created using the Fast Fourier Transform of all speech files. The attenuation rate of the SSN was 12 dB/octave at 1000 Hz. Both noises had similar frequency ranges and long-term-average-speech spectra as the sentences and were normalized to have similar root mean square (RMS) amplitudes.

#### 2.2.5. Sentences for Children

This included eight lists of 20 sentences each, which were taken from a pool of sentences in Hebrew created according to the hearing-in-noise test (HINT) criteria [18]. The sentences in each list were equal in length (five to nine syllables per sentence) and presented at 65 dB SPL by a recorded female voice. The test was conducted in quiet conditions for all children.

### 2.3. Design and Procedure

All participants were tested individually in a quiet room, at a distance of one meter from a loudspeaker. Upon arrival, all participants received an explanation of the experimental tasks. During the tests, CI users used their devices with the program and setting they preferred for everyday use. If participants wore a contralateral hearing aid, they were allowed to wear it during testing. Demographic and background data were obtained (via questionnaire) and the following tests were performed: Children were evaluated on the Hebrew DIN test, as well as HAB and sentences in quiet tests. Adults were evaluated on the Hebrew DIN test, HAB in quiet and noise tests, and HeBio sentences in quiet and noise tests. The HeBio sentences in the quiet and in noise tests were delivered using the Advanced Bionics ListPlayer software on a Lenovo laptop computer via a Genelec 810A loudspeaker and a MAYA 44 external sound card. For the adaptive procedure, the ListPlayer software controlled the noise level according to the relevant SNRs. The sentence intensity was calibrated to 65 dB SPL at the participant’s location using a sound-level meter with a calibration tone of 1000 Hz. The HAB and sentences for the children were delivered from the same laptop, loudspeaker, and external sound card. The Hebrew DIN was delivered via an I-PAD connected directly to a Genelec 810A loudspeaker. The calibration of the intensity was performed using a sound-level meter. In the NH group, pure-tone audiometric thresholds were assessed using an Interacoustics-AD629 audiometer with headphones. The full experimental session lasted no more than one hour, and breaks were offered upon the participants’ request.

### 2.4. Statistical Analysis

A statistical analysis was conducted using SPSS Statistics 26, IBM Corp (Armonk, NY, USA). Age (children, adults) and hearing status (NH, CI users) effects were analyzed using univariate ANOVA. Among the CIs, independent-sample *t*-tests were used to test the age differences between children and prelingual adults, and the onset-of-deafness differences between pre- and post-lingual adults. Participants in different age groups performed tests of speech perception appropriate to their respective levels to avoid ceiling/floor effects. Therefore, comparisons between hearing status in these tests, as well as correlations between them, were conducted separately for each age group, using MANOVA and Pearson correlation.

## 3. Results

### 3.1. Hebrew DIN in CI Users and NH Aged-Matched Controls

#### 3.1.1. Age and Hearing Status

**Figure 1:**
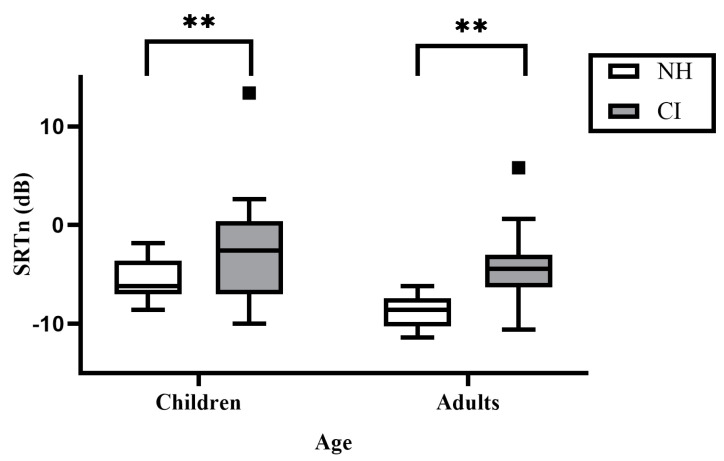
Box-and-whisker plot of Hebrew digits in noise (DIN) speech receptive thresholds in noise (SRTn) (dots beyond the whiskers symbolize outliers) for CI users (children, and pre- and post-lingual adults) and NH matched groups. CI, cochlear implants; NH, normal hearing; SRTn, speech reception thresholds in noise. Higher SRTn means worse performance. ** *p* < 0.01.

Main effects were observed for both age (F(1,87) = 17.371, *p* < 0.001, partial η^2^ = 0.173) and hearing status (F(1,87) = 29.362, *p* < 0.001, partial η^2^ = 0.261), but not for hearing status × age interaction (F(1,87) = 0.121, *p* = 0.729, partial η^2^ = 0.001). Figure 1 presents the mean SRTn for each of the four groups. All CI users had higher SRTn (mean = −3.160, SE = 0.542) than their NH peers (mean = −7.160, SE = 0.502). The children had a higher SRTn (mean = −3.622, SE = 0.614) than the adults (mean = −6.698, SE = 0.409).

#### 3.1.2. Age and Onset-of-Deafness

**Figure 2:**
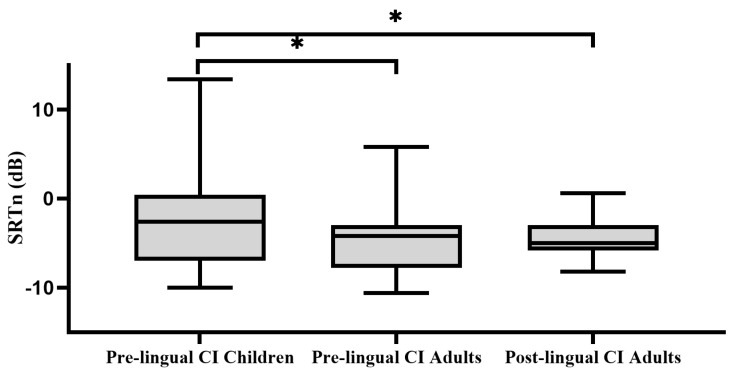
Box-and-whisker plot of Hebrew digits in noise (DIN) speech receptive thresholds in noise (SRTn) for three groups of CI users (pre-lingual CI children, pre-lingual CI and post-lingual CI adults users). CI, cochlear implants; NH, normal hearing; SRTn, speech reception thresholds in noise. Higher SRTn means worse performance. * *p* < 0.05.

Figure 2 presents Hebrew DIN SRTn for the three CI groups. Pre-lingual children had a higher Hebrew DIN SRTn than pre-lingual adults (t(26) = −1.966, *p* = 0.03). Pre-lingual children also had a higher Hebrew DIN SRTn than the post-lingual adults (t(26) = −1.637, *p* = 0.05). No difference was found between pre- and post-lingual adults (t(28) = 0.736, *p* = 0.766).

### 3.2. Hebrew DIN and Other Speech Perception Tests

#### 3.2.1. Children

**Figure 3:**
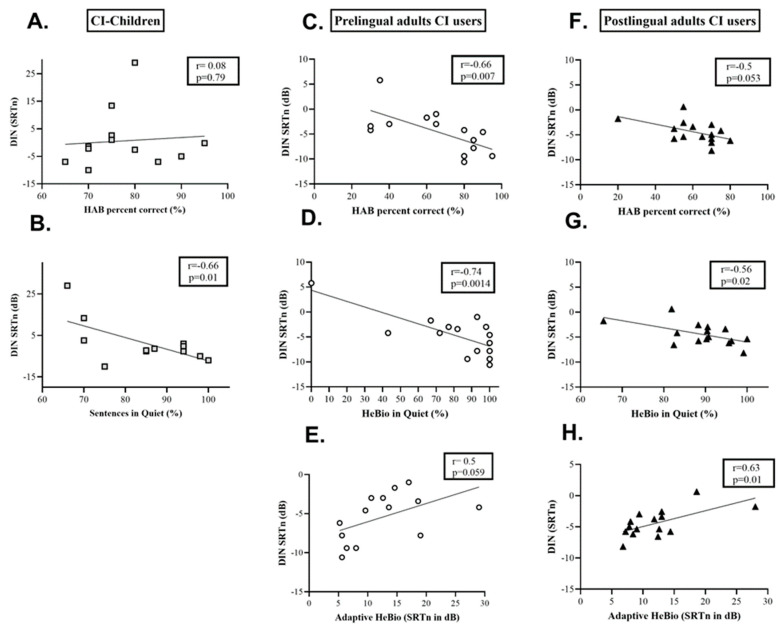
Correlations between speech receptive thresholds in noise (SRTn) on the Hebrew digits in noise (DIN) and results in speech perception tests for the CI study groups: (**A**) percentage of correct correct words on HAB word recognition test in quiet for children with CIs; (**B**) percentage of sentences in quiet for children with CIs, (**C**) percentage of correct words on HAB word recognition test in quiet for pre-lingual CI adults users, (**D**) percentage of HeBio sentences in quiet tests for pre-lingual CI adults users, (**E**) SRTn for adaptive HeBio sentences for pre-lingual CI adults users, (**F**) percentage of correct words on HAB word recognition test in quiet for postlingual CI adults users, (**G**) percentage of HeBio sentences in quiet tests for postlingual CI adults users, (**H**) SRTn for adaptive HeBio sentences for postlingual CI adults users. Hebrew DIN, digits in noise; SRTn, speech reception thresholds in noise, HAB, Hebrew Arthur Boothroyd. The lines on the figures represent linear regression trend lines.

A significant main effect was found for hearing status (F(3,23) = 17.849, *p* < 0.001, partial η^2^ = 0.700). Table 1 section A shows that children with CIs had a higher Hebrew DIN SRTn and lower word and sentence accuracy than NH children. No correlation was found between the Hebrew DIN SRTn and HAB accuracy (Figure 3A). A significant negative association was found between the Hebrew DIN SRTn and sentence accuracy in the quiet test for children with CIs (Figure 3B).

#### 3.2.2. Pre-Lingual CI Users Adults

**Figure 4:**
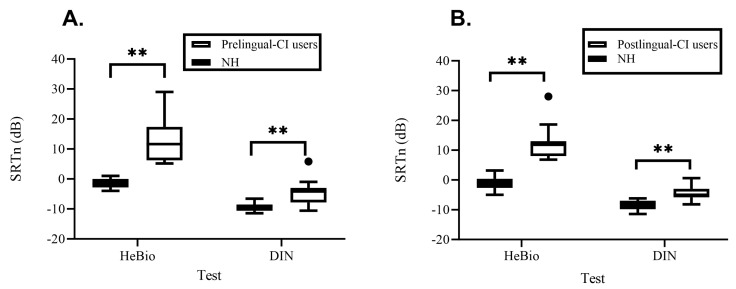
Box-and-whisker plot of the SRTn for the adaptive HeBio sentence test and the SRTn for the DIN test (dots beyond the whiskers symbolize outliers), for pre-lingual CI users (white) and NH adults (black) (**A**) and for post-lingual CI users (white) and NH adults (black) (**B**). CI, cochlear implants; NH, normal hearing; SRTn, speech reception thresholds in noise. ** *p* < 0.01.

A significant main effect was found for the hearing status (F(2,25) = 31.338, *p* < 0.001, partial η^2^ = 0.715). Table 1 section B and Figure 4A show that young adults with pre-lingual CIs had higher Hebrew DIN and HeBio SRTn values than young adults with NH.

Significant negative associations were found between the Hebrew DIN SRTn values and the accuracy of the HAB and Hebio in quiet tests (Figure 3C,D). A marginally significant correlation was found between the Hebrew DIN test and adaptive HeBio in noise SRTn (Figure 3E).

#### 3.2.3. Post-Lingual CI Adults Users

A significant main effect was found for the hearing status (F(2,27) = 35.033, *p* < 0.001, partial η^2^ = 0.722). Table 1 section C and Figure 4B show that older adults with post-lingual CI had higher Hebrew DIN and HeBio SRTn values than NH older adults. A significant negative association was found between Hebrew DIN SRTn and HeBio in quiet test accuracy, and a significant positive association was found between the SRTn of Hebrew DIN and adaptive HeBio in noise (Figure 3G,H). A marginally significant correlation was found between Hebrew DIN SRTn and HAB in quiet test accuracy (Figure 3F).

## 4. Discussion

In the current study, we examined the Hebrew DIN test thresholds in groups of CI users in different age groups, along with different onsets of deafness (pre-lingual versus post-lingual deafness) and their NH peers. As expected, we found that the Hebrew DIN SRTn was higher among all the CI groups than among their NH peers. There was also an effect of age, as children had a higher SRTn than adults, but there was no difference between pre-lingual CI and post-lingual CI adults. Finally, we observed a significant correlation between Hebrew DIN scores and most speech perception tests.

The finding that children (both NH and CI users) achieved a higher Hebrew DIN SRTn than adults is supported by previous studies showing that children struggle more than adults in noisy environments and require more favorable SNRs to perform as well as adults [19,20]. Also, the finding that CI users required a higher mean Hebrew DIN SRTn than NH individuals is supported by previous studies [12,13,14]. Nevertheless, in more difficult tests, such as sentences in noise (HeBio), the difference in SRTn between CI adult users and their NH peers was much larger (mean of 13–14 dB as opposed to 3–5 dB in the DIN test). Additionally, approximately half of the children using CIs and 20–33% of the adult CI groups had Hebrew DIN SRTn within the range of their NH peers, and no overlap was observed in the more challenging sentences in the noise (HeBio) test. Therefore, although the Hebrew DIN test differentiates between CI users and NH peers, its discerning is insufficient. Additional tests with a range of listening conditions and linguistic redundancies are warranted to fully assess the speech perception abilities of CI users in daily life.

Post-lingual CI users develop linguistic skills through full auditory input, whereas pre-lingual users rely on degraded auditory stimuli via their implant devices. Consequently, post-lingual CI users were hypothesized to have better SRTn than pre-lingual CI users. However, the results did not support this hypothesis, as there was no significant difference in the mean Hebrew DIN SRTn between these groups. One possible explanation is that the DIN test may not be sufficiently sensitive to measure the effects of early auditory exposure. Another explanation is that the large variability in the results of the pre-lingual CI users, with some individuals receiving very poor results (positive SRTn) and others receiving normal thresholds, may have blurred the differences between the two groups. This also suggests that factors other than auditory exposure (e.g., duration CI usage, auditory training, etc.) may play a role in speech perception abilities.

Marginal to significant associations were found between the Hebrew DIN test and other standard speech perception tests among CI users. This finding is consistent with other studies that have shown correlations between the DIN test and standard speech perception tests using words and sentences [13,14]. The high correlation observed underscores the DIN test’s utility in comprehensively assessing speech perception abilities in CI users. Furthermore, the alignment between the DIN test outcomes and those of traditional speech perception tests reinforces the validity of using the DIN test as a complementary tool in clinical assessments.

The DIN test was originally developed for hearing screening [11,21]. Nevertheless, it has been increasingly used as a part of speech perception evaluation battery for CI users [22]. Therefore, it is important to note that the DIN test alone is insufficient for a comprehensive evaluation of both pre- and post-implantation, and other speech perception tests in both quiet and noisy environments should be incorporated. A notable application of the DIN is its use as a remote assessment to gauge CI user function by CI device manufacturers. This practice significantly benefits CI users by reducing the need for routine visits to distant CI centers. However, it is important to remember that while valuable, this method provides a relatively nonspecific measure of function.

The relatively limited number of participants in each group may lead to biased results. However, this sample size is accepted in relevant studies [22,23]. Future research may benefit from replicating this study with larger groups. Additionally, not all tests were administered to both children and adults, as some tests are not suited to the linguistic abilities and working memory capacity of children. Longitudinal studies can track changes in speech perception in noise over time with CI use. The current study compared children and adults, and adding a comparison between young and older adults can expand the results of the existing findings.

## 5. Conclusions

The results of the present study indicate that the Hebrew DIN test is an efficient and rapid measure for evaluating speech perception under noisy conditions with minimal linguistic requirements. It is highly correlated with other speech perception measures and can differentiate between CI users and NH controls, although it may not be sufficiently sensitive to differentiate between CI users with different onsets of deafness. The Hebrew DIN test is sensitive to age differences, with children performing worse than adults in both the NH and CI groups. Taking these characteristics together, we recommend the Hebrew DIN test as an additional test to the standard battery of speech perception tests for both CI candidates and users.

## Figures and Tables

**Table 1:** Mean (standard deviations, SD) and results of univariate ANOVAs (F, *p*, and partial η^2^) between cochlear implant (CI) and normal hearing (NH) children (A), pre-lingual CI adult users (B), and post-lingual CI older adult users (C) in Hebrew digits in noise (DIN) and speech perception tests.

	CI	NH	F (1,27)	*p*	Partial η^2^
A. Children					
DIN_SRTn	−1.75 (5.98)	−5.49 (2.18)	5.08	0.033	0.17
HAB_quiet	0.78 (0.09)	0.97 (0.04)	52.87	<0.001	0.68
Sentences_quiet	0.88 (0.10)	0.97 (0.09)	5.31	0.030	0.18
B. Pre-lingual CI adult users					
DIN_SRTn	−5.15 (2.98)	−9.29 (1.37)	23.44	<0.001	0.47
HeBio_SRTn	12.88 (6.91)	−1.55 (1.68)	61.47	<0.001	0.70
C. Post-lingual CI adult users					
DIN_SRTn	−4.44 (2.19)	−8.36 (1.53)	32.26	<0.001	0.54
HeBio_SRTn	12.03 (5.48)	−0.99 (2.24)	72.53	<0.001	0.72

## Data Availability

The raw data for this study are available from the corresponding author on reasonable request.

## Appendix A

**Table A1:** Demographic information and background data for the three CI groups.

No.	Age (y)	Gender	No. of CIs	Implanted Ear	Age at Onset	Age at 1st CI	Age at 2nd CI	Duration Implant Use (y)	HA Use	Type of CI	Etiology
Pre-lingual CI Children											
1	7.8	M	1	L	2.5	6.5	--	5.3	Y	AB	Unknown
2	5.9	M	1	L	1.2	1.5	--	4.4	Y	AB	Meningitis
3	7.3	F	1	L	0	0.9	--	6.4	Y	C	CMV
4	6.5	F	2	R, L	0	0.75	0.75	5.75	AB	Genetic
5	6.5	M	2	R, L	0	1.4	1.4	5.1	AB	Unknown
6	6.4	F	2	R, L	0	0.9	0.9	5.5	C	Unknown
7	7.4	F	1	R	0	3.8	--	3.6	Y	AB	Unknown
8	6.6	M	2	R, L	1.2	1.5	1.5	5.1	C	Infection
9	7.9	M	2	R, L	0	0.9	0.9	7	C	Genetic
10	5.5	M	2	R, L	2.3	3.1	3.1	2.4	C	Meningitis
11	10.5	F	2	R, L	0	1.1	1.1	9.4	C	Unknown
12	9.6	M	1	L	0	4.5	--	5.1	Y	C	CMV
13	11	F	2	R, L	0	1	4	7		C	Unknown
Pre-lingual CI adults users											
1	25.5	F	2	R, L	1.5	4	14	21.5	C	CMV
2	23	F	2	R, L	0	2	13	21	C	Familial
3	23	F	2	R, L	0	2	14	21	C	Familial
4	28	M	1	R	0	6	--	22	C	Unknown
5	30	F	2	R, L	0	3	14	27	C	Genetic
6	27	M	2	R, L	0	3	26	24	C	Genetic
7	28.5	F	1	R	0	8	--	20.5	Y	AB	Genetic
8	16	F	2	R, L	0	1	10	15	C	Unknown
9	16	M	2	R, L	0	1	6	15	C	Unknown
10	26	M	2	R, L	0	4	25	22	C	Unknown
11	30	F	2	R, L	0	3	14	27	C	Familial
12	20	F	2	R, L	0	3	7	17	C	Unknown
13	23	F	2	R, L	0	2	11	21	C	Unknown
14	15	F	2	R, L	0	1	4	14	C	Genetic
15	14	M	2	R, L	0	1.25	1.25	12.55		C	Genetic
Post-lingual CI adults users											
1	36	F	1	L	15	31	--	5	AB	Unknown
2	49	F	1	L	32	40	--	9	Y	M	Otosclerosis
3	60	F	1	L	10	45	--	15	C	Genetic
4	22	F	1	R	14	17	--	3	M	Unknown
5	24	F	2	R, L	8	19	21	5	AB	Unknown
6	68	F	1	R	50	64	--	4	Y	C	Genetic
7	55	F	1	L	35	45	--	10	Y	C	Meniere
8	56	F	2	R, L	18	51	54	5	M	Unknown
9	62	M	2	R, L	35	59	60	3	M	Otosclerosis
10	29	F	1	L	10	28	--	1	Y	AB	Meningitis
11	62	M	1	L	40	58	--	4	Y	M	Otosclerosis
12	77	F	1	R	47	73	--	4	Y	C	Genetic
13	74	F	1	R	57	72	--	2	Y	AB	Unknown
14	60	F	1	L	38	58	--	2	Y	C	Autoimmune
15	32	F	1	R	10	31	--	1	Y	C	Unknown

No. = number, (y) = years; Gender: F = female, M = male; Implanted Ear: R = right, L = left; HA = hearing aid; CI = cochlear implant; Type of CI: C = Cochlear, AB = Advanced Bionics, M = Med-EL, CMV = Cytomegalovirus.
